# The Indolinone MAZ51 Induces Cell Rounding and G2/M Cell Cycle Arrest in Glioma Cells without the Inhibition of VEGFR-3 Phosphorylation: Involvement of the RhoA and Akt/GSK3β Signaling Pathways

**DOI:** 10.1371/journal.pone.0109055

**Published:** 2014-09-30

**Authors:** Joo-Hee Park, Yoo-Jin Shin, Tae-Ryong Riew, Mun-Yong Lee

**Affiliations:** Department of Anatomy, Catholic Neuroscience Institute, College of Medicine, The Catholic University of Korea, Seoul, Korea; University of Montréal and Hôpital Maisonneuve-Rosemont, Canada

## Abstract

MAZ51 is an indolinone-based molecule originally synthesized as a selective inhibitor of vascular endothelial growth factor receptor (VEGFR)-3 tyrosine kinase. This study shows that exposure of two glioma cell lines, rat C6 and human U251MG, to MAZ51 caused dramatic shape changes, including the retraction of cellular protrusions and cell rounding. These changes were caused by the clustering and aggregation of actin filaments and microtubules. MAZ51 also induced G2/M phase cell cycle arrest. This led to an inhibition of cellular proliferation, without triggering significant cell death. These alterations induced by MAZ51 occurred with similar dose- and time-dependent patterns. Treatment of glioma cells with MAZ51 resulted in increased levels of phosphorylated GSK3β through the activation of Akt, as well as increased levels of active RhoA. Interestingly, MAZ51 did not affect the morphology and cell cycle patterns of rat primary cortical astrocytes, suggesting it selectively targeted transformed cells. Immunoprecipitation–western blot analyses indicated that MAZ51 did not decrease, but rather increased, tyrosine phosphorylation of VEGFR-3. To confirm this unanticipated result, several additional experiments were conducted. Enhancing VEGFR-3 phosphorylation by treatment of glioma cells with VEGF-C affected neither cytoskeleton arrangements nor cell cycle patterns. In addition, the knockdown of VEGFR-3 in glioma cells did not cause morphological or cytoskeletal alterations. Furthermore, treatment of VEGFR-3-silenced cells with MAZ51 caused the same alterations of cell shape and cytoskeletal arrangements as that observed in control cells. These data indicate that MAZ51 causes cytoskeletal alterations and G2/M cell cycle arrest in glioma cells. These effects are mediated through phosphorylation of Akt/GSK3β and activation of RhoA. The anti-proliferative activity of MAZ51 does not require the inhibition of VEGFR-3 phosphorylation, suggesting that it is a potential candidate for further clinical investigation for treatment of gliomas, although the precise mechanism(s) underlying its effects remain to be determined.

## Introduction

Indolinones, bearing different amino acid moieties in the 3 position, are a class of ATP-competitive receptor tyrosine kinase inhibitors that have proven to be selective for certain receptor tyrosine kinases, including receptors for vascular endothelial growth factor (VEGF), epidermal growth factor, fibroblast growth factor, and platelet-derived growth factor [1]. These small molecules are being extensively studied for their biological activities in several pharmaceutical areas. Several indolinone derivatives show antiproliferative activity in cancer cell lines by inhibiting various kinase families. This activity suggests the potential therapeutic application of indolinones as antitumor agents [1]–[7]. Of particular interest has been MAZ51 (3-(4-dimethylamino-naphthelen-1-ylmethylene)-1,3-dihydroindol-2-one), an indolinone-based synthetic molecule that potently inhibits both VEGF-C-dependent and VEGF-C-independent VEGF receptor (VEGFR)-3 phosphorylation in endothelial cell lines [2], [8], [9]. MAZ51 also has anti-angiogenic effects and directly suppresses tumor cell growth and progression in rat carcinoma cells and in oral squamoid cancer cells via inhibition of the VEGF-C/VEGFR-3 axis [3], [8], [10].

Glioblastoma is the most common and malignant primary brain tumor and is characterized by its metastatic dissemination and poor prognosis [11], [12]. Although the actions of VEGF-C/VEGFR-3 have been studied extensively in the lymphatic system [13], [14], VEGF-C and VEGFR-3 are also expressed in glioblastomas and hemangioblastomas that are devoid of lymphatic vessels [15]. In addition, upregulation of VEGFR-3 is observed in glioblastomas, compared to low-grade gliomas and non-neoplastic brain tumors, indicating that expression of VEGFR-3 in gliomas correlates with the tumor grade [16]. Thus, we speculate that MAZ51 might have antitumor activity in gliomas by inhibiting VEGFR-3 signaling. The current study was designed to examine the *in* *vitro* effects of MAZ51 in rat C6 glioma cells, which share a number of characteristics with human glioblastoma cells [17], and in the U251MG human glioma cell line. In parallel, we used rat primary cortical astrocytes as a non-transformed model of glial cells.

In this study, we demonstrate that MAZ51 causes dramatic cellular morphological changes by altering the cytoskeleton and inducing cell cycle arrest at G2/M in glioma cells, but not in primary cortical astrocytes. We also provide evidence that phosphorylation of Akt/GSK3β and activation of RhoA are involved in the effects of MAZ51. Unexpectedly, MAZ51 did not inhibit tyrosine phosphorylation of VEGFR-3 in glioma cells. This unanticipated result indicated that the antitumor activity of MAZ51 in gliomas is likely to be independent of its inhibition of VEGFR-3 phosphorylation, although the precise mechanism remains to be determined.

## Materials and Methods

### Cell culture

The C6 rat glioma cell line was obtained from the Korean Cell Line Bank (Seoul, Korea). The U251MG human glioma cell line was provided by St. Mary’s Hospital, Department of Neurosurgery Laboratory (Seoul Korea). The cells were grown and maintained in Dulbecco’s Modified Eagle’s Medium (DMEM, Gibco BRL, CA, USA) containing 50 U/ml penicillin/streptomycin (Biowest, Nuaillé, France) and supplemented with 10% heat-inactivated fetal bovine serum (FBS, Gibco). Cells were incubated at 37°C under 5% CO_2_.

Rat primary cortical astrocytes were isolated from 1-day old Sprague Dawley rat pups. The cerebral cortices were aseptically dissected, and tissues were placed in Hank's Balanced Salt Solution (HBSS) containing 0.25% trypsin-EDTA (Biowest). Cortical astrocytes were dissociated for 15 min using a Pasteur pipette, then kept at 37°C for 10 min and centrifuged at 400 *g* for 5 min. The pellet was re-suspended in DMEM and gently dissociated. After another centrifugation step (400 *g*, 5 min), the cells were re-suspended in DMEM and centrifuged for 7 min (400 *g*). The pelleted cells were re-suspended in DMEM in 185 cm^2^ tissue culture flasks and incubated at 37°C under 5% CO_2_.

Animal care was conducted in accordance with the Laboratory Animal Welfare Act, the Guide for the Care and Use of Laboratory Animals, and the Guidelines and Policies for Rodent Survival Surgery provided by the IACUC (Institutional Animal Care and Use Committee) of the College of Medicine, at the Catholic University of Korea.

### Immunocytochemistry

Cells were seeded at a density of 4×10^4^ cells per milliliter and grown on 4-well Lab-Tek chamber slides (Nalgene Nunc Penfield, NY, USA). Cells were fixed with absolute ethanol for 10 min and blocked using 5% bovine serum albumin in Dulbecco’s phosphate-buffered saline (DPBS, Biowest). The chamber slides were incubated overnight at 4°C with mouse monoclonal anti-alpha tubulin (1∶8000; Sigma-Aldrich, St. Louis, MO, USA). The chamber slides were then washed with DPBS (Biowest) and incubated with Cy3-conjugated anti-mouse antibody (1∶2000; Jackson ImmunoResearch, West Grove, PA, USA) for 2 h at room temperature. Staining for F-actin was carried out using tetramethylrhodamine isothiocyanate (TRITC)-conjugated phalloidin (1 µg/mL; Sigma-Aldrich). Counterstaining of cell nuclei was carried out using 4′,6-diamidino-2′-phenylindole (DAPI, Roche, Germany; dilution 1∶1000) for 10 min at room temperature. Slides were viewed using a confocal microscope (LSM 700; Carl Zeiss Co. Ltd., Oberkochen, Germany).

### Cell cycle analysis

Cells were cultured overnight on 6 well plates and then incubated for 24 h either in the absence or in the presence of MAZ51 (Calbiochem, San Diego, CA, USA). The concentration of MAZ51 used was based on previous studies [2], [18]. MAZ51 was dissolved in DMSO and added to cells at 2.5 or 5 µM. The final DMSO concentration was 0.1% (v/v) and control cells were treated with this amount of DMSO alone. For bromodeoxyuridine (BrdU) analyses, cells were pulsed with 1 mM BrdU and processed with the BrdU Flow Kit (BD Pharmingen, San Diego, CA, USA) according to the manufacturer’s instructions. Briefly, BrdU-pulsed cells were fixed and permeabilized, then washed with BD washing buffer. After re-fixation and DNase treatment, the fluorescein isothiocyanate (FITC)-conjugated anti-BrdU antibody was added for 20 min at room temperature. Cells were re-suspended in 20 µL of the 7-amino-actinomycin D (7-AAD) solution to stain total DNA for cell cycle analysis, and then re-suspended in BD Pharmingen stain buffer. The cell cycle profiles were analyzed by FACScan flow cytometry (Becton Dickinson, San Jose, CA, USA). Statistical significance was determined using two-way ANOVA followed by the Bonferroni multiple comparison test. *P*<0.05 was regarded as significant using GraphPad Prism Ver. 5.01 (GraphPad Software Inc., La Jolla, CA, USA).

### Immunoprecipitation and western blot analysis

For the immunoprecipitation studies, C6 glioma cells were used untreated, treated with medium containing 0.1% (v/v) DMSO, or treated with medium containing 2.5 or 5 µM concentrations of MAZ51. Cells were collected at 6 and 24 h after MAZ51 treatment. In addition, cells were treated with 150 ng/ml recombinant rat VEGF-C protein (ReliaTech GmbH, Wolfenbuttel, Germany) for 24 h. Identical amounts of protein from each sample were resuspended with protein G-Sepharose (Sigma-Aldrich) and 1 µg of normal rabbit IgG (Santa Cruz, CA, USA) for 1 h at 4°C. Following the removal of protein G-Sepharose by brief centrifugation (6,000 *g*), the supernatant was incubated with 1 µg of Flt4 antibody (Santa Cruz) for 1 h at 4°C. Immunoprecipitation of the antibody-antigen complexes was performed by incubation overnight at 4°C with 80 µL of protein G-Sepharose. Nonspecifically bound proteins were removed by washing the Sepharose beads three times with lysis buffer and one time with DPBS. Bound proteins were solubilized in 50 µL of 2×Laemmli sodium dodecyl sulfate (SDS) sample buffer and further analyzed by western blotting. Samples were separated by SDS-polyacrylamide gel electrophoresis (SDS-PAGE, 6%), and transferred to PVDF membranes. Nonspecific binding was blocked with 2.5% BSA in TTBS (10 mM Tris, pH 8.0, 150 mM NaCl, 0.1% Tween 20) and samples were incubated with the anti-phosphotyrosine antibody PY99 (1∶500; Santa Cruz) at 4°C overnight. Immunoreactive bands were visualized using a peroxidase-conjugated secondary antibody and an enhanced chemiluminescence (ECL) kit (Amersham, GE healthcare, UK). Each experiment was repeated at least 3 times, and representative blots are shown.

### Rho pull-down assay

RhoA activation was examined using the Active-Rho Pull-Down and Detection Kit (Pierce, Rockford, IL, USA). Cells were lysed in buffer containing 25 mM Tris–HCl (pH 7.2), 150 mM NaCl, 1% NP-40, 5% glycerol and 5 mM MgCl_2_. The protein concentration of cell extracts was determined by the Bradford assay (Bio-Rad, Hercules, CA, USA). Five hundred micrograms of protein were incubated with glutathione S-transferase (GST)-Rhotekin-Rho-binding domain (RBD) immobilized on a glutathione resin slurry at 4°C for 1 h. The resin was then washed twice with lysis buffer. Guanosine triphosphate (GTP)-bound RhoA was released from the resin by heating at 95°C for 5 min in 2×Laemmli SDS-sample buffer. The RhoA proteins were then resolved by 12% SDS–PAGE and detected by western blot using the kit containing the RhoA antibody (1∶300). Active GTP-bound RhoA was normalized against total RhoA using the same amount of cell lysate.

### Western blot analysis

Cells were lysed in ice-cold RIPA buffer (50 mM Tris-HCl (pH 8.0), 150 mM NaCl, 1% NP-40, 0.5% deoxycholate, and 0.1% SDS) for 30 min. The debris was removed by centrifugation at 16,000 *g* for 1 min. Equal amounts (30 µg) of total cell protein were separated by SDS-PAGE (10%), and transferred to the PVDF membrane. After blocking with 5% BSA in TTBS buffer for 1 h at room temperature, membranes were incubated overnight at 4°C with the following primary antibodies: rabbit anti-GSK3β (1∶1000; Cell Signaling, Beverly, MA, USA), rabbit anti-pGSK3β (1∶1000; Cell Signaling), rabbit anti-Akt (1∶1000; Cell Signaling), rabbit anti-pAkt (1∶1000; Cell Signaling), rabbit anti-Flt4 (1∶500; Santa Cruz), anti-Rho (1∶5000; Santa Cruz), and β-actin (1∶10000; Sigma-Aldrich). The membranes were incubated with peroxidase-conjugated secondary antibody for 1 h at room temperature. Blots were developed using an ECL kit (Amersham, GE Healthcare, UK). Each experiment was repeated at least three times, and the densitometric analysis was performed using Multi Gauge V3.0 software (Fujifilm Life Science, Tokyo, Japan). Statistical significance was determined using one-way ANOVA followed by the Bonferroni multiple comparison test. *P*<0.05 was regarded as significant using GraphPad Prism Ver. 5.01.

### Small interfering RNA (siRNA) transfection

To decrease the expression of VEGFR-3, siRNA duplexes specific for rat VEGFR-3 were purchased from Bioneer (Daejeon, Korea). Target sequences of siRNA duplexes were as follows: sense (5′-GCAAACUUCGCUACACUAA (dTdT)-3′), and antisense (5′-GGUUGCCAGUUAUAGGUAU (dTdT)-3′). Transient transfection of VEGFR-3 siRNA into C6 glioma cells was carried out using Lipofectamine RNAiMAX (Invitrogen, CA, USA) according to manufacturer’s protocol. Knockdown efficiency was evaluated 72 h after transfection by measuring VEGFR-3 protein levels in cell lysates using western blot analysis. Transfected cells were then fixed with absolute ethanol for 10 min and used in immunocytochemistry assays and F-actin staining as detailed above.

### Quantitative real-time reverse transcriptase–polymerase chain reaction (qRT–PCR) and electrophoresis

Total RNA was isolated from cultured cells by using TRIzol (Invitrogen), and the purity and yield of the RNA were determined spectrophotometrically. One microgram of total RNA from each sample was reverse transcribed into cDNA by using the RevertAid First Strand cDNA Synthesis kit (Thermo Scientific, IL, USA) in a total volume of 20 µL, according to the manufacturer’s instructions. PCR amplification was performed using 100 ng of cDNA from each RNA sample, specific oligonucleotide primers (Figure S1), and a PCR Thermal Cycler Dice (Takara Bio, Shiga, Japan). PCR conditions were as follows: 35 cycles of 30 s at 94°C, 30 s at 54°C, and 45 s at 72°C. PCR products were resolved on 0.8% agarose gels stained with Loading STAR (Dyne Bio, Seoul, Korea). qRT-PCR was performed using 500 ng of cDNA as a template and the SYBR Green Premix Ex Taq kit (Takara Bio) on an ABI ViiA 7 Real-Time PCR System (Applied Biosystems, Carlsbad, CA, USA). The qRT-PCR primer sequences are shown in Figure S1, and dissociation curves were generated to check the specificity of the primer annealing to the template. The expression level of VEGFR-3 was calculated using the comparative threshold cycle method (2^−ΔΔCt^) with *GAPDH* as the control gene. All PCR assays were performed in triplicate. Statistical significance was determined using one-way ANOVA followed by the Bonferroni multiple comparison test. *P*<0.05 was regarded as significant using GraphPad Prism Ver. 5.01.

## Results

### MAZ51 reversibly alters the organization of F-actin and microtubules, inducing morphological changes in glioma cell lines but not in primary astrocytes

**Figure 1:**
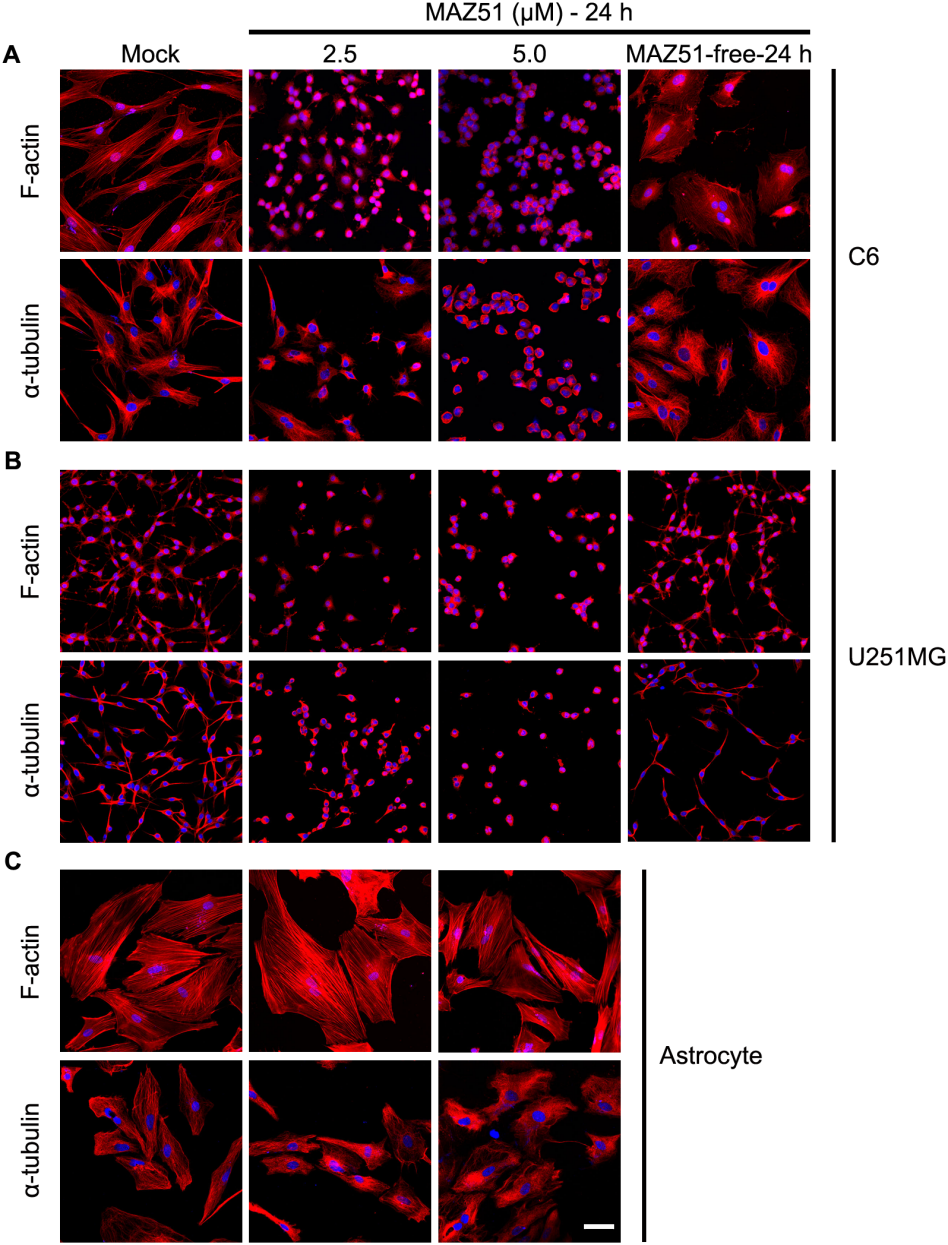
MAZ51 reversibly alters the cytoskeletal organization in glioma cells but not in primary astrocytes. Two glioma cell lines, rat C6 (A) and human U251MG (B), and primary rat cortical astrocytes (C) were treated for 24 h either in the absence (control) or presence of MAZ51 (2.5 µM or 5.0 µM). Glioma cells were also treated with 5.0 µM MAZ51 for 24 h and then incubated for an additional 24 h in fresh medium without MAZ51 (MAZ51-free-24 h). Morphological changes and cytoskeletal rearrangements were assessed by staining with TRITC-labeled phalloidin for filamentous actin (F-actin) or by immunocytochemistry using an anti-α-tubulin antibody for microtubules. Cell nuclei appear blue after DAPI staining. Data are representative of three independent experiments. Scale bar = 50 µm.

Dramatic morphological transformation was observed in two different glioma cell lines, C6 and U251MG, at an MAZ51 concentration as low as 2.5 µM. This effect was pronounced at 5.0 µM, a dose shown to inhibit VEGFR-3 activity [2]. Because the cytoskeleton, including the actin and microtubule networks, is critical for regulating cell shape, more detailed analyses of the effects of MAZ51 on the structural changes in the cytoskeleton were performed using TRITC-labeled phalloidin and an anti-α-tubulin antibody, which are well-established markers of F-actin and microtubule stability, respectively (Fig. 1). In the absence of MAZ51 treatment, C6 cells showed a flattened and polygonal morphology with well-organized actin stress fibers and microtubules distributed in all of the cytoplasmic compartments (Fig. 1A). However, cells treated with MAZ51 showed extensive clustering and aggregation of F-actin and microtubules, inducing the retraction of cellular protrusions and cell rounding (Fig. 1A). The cell rounding was observed within 1 h after MAZ51 treatment (data not shown), and virtually all cells were rounded at 24 h. These morphological changes were also observed when U251MG cells were treated with MAZ51 (Fig. 1B). Interestingly, the rounded MAZ51-treated cells rapidly reacquired their original morphology when MAZ51-containing medium was replaced with fresh medium without MAZ51 (Figs. 1A, B), suggesting that the MAZ51-induced cytoskeletal alteration is reversible.

We then investigated whether the shape changes observed in MAZ51-treated glioma cells also occurred in cultured primary astrocytes. Unlike in glioma cell lines, MAZ51 caused no appreciable changes in cell shape or cytoskeleton arrangements (affecting F-actin and microtubules) in these cells (Fig. 1C).

### MAZ51 reversibly causes cell cycle arrest at the G2/M phase in glioma cell lines, but not in primary astrocytes, without triggering cell death

**Figure 2:**
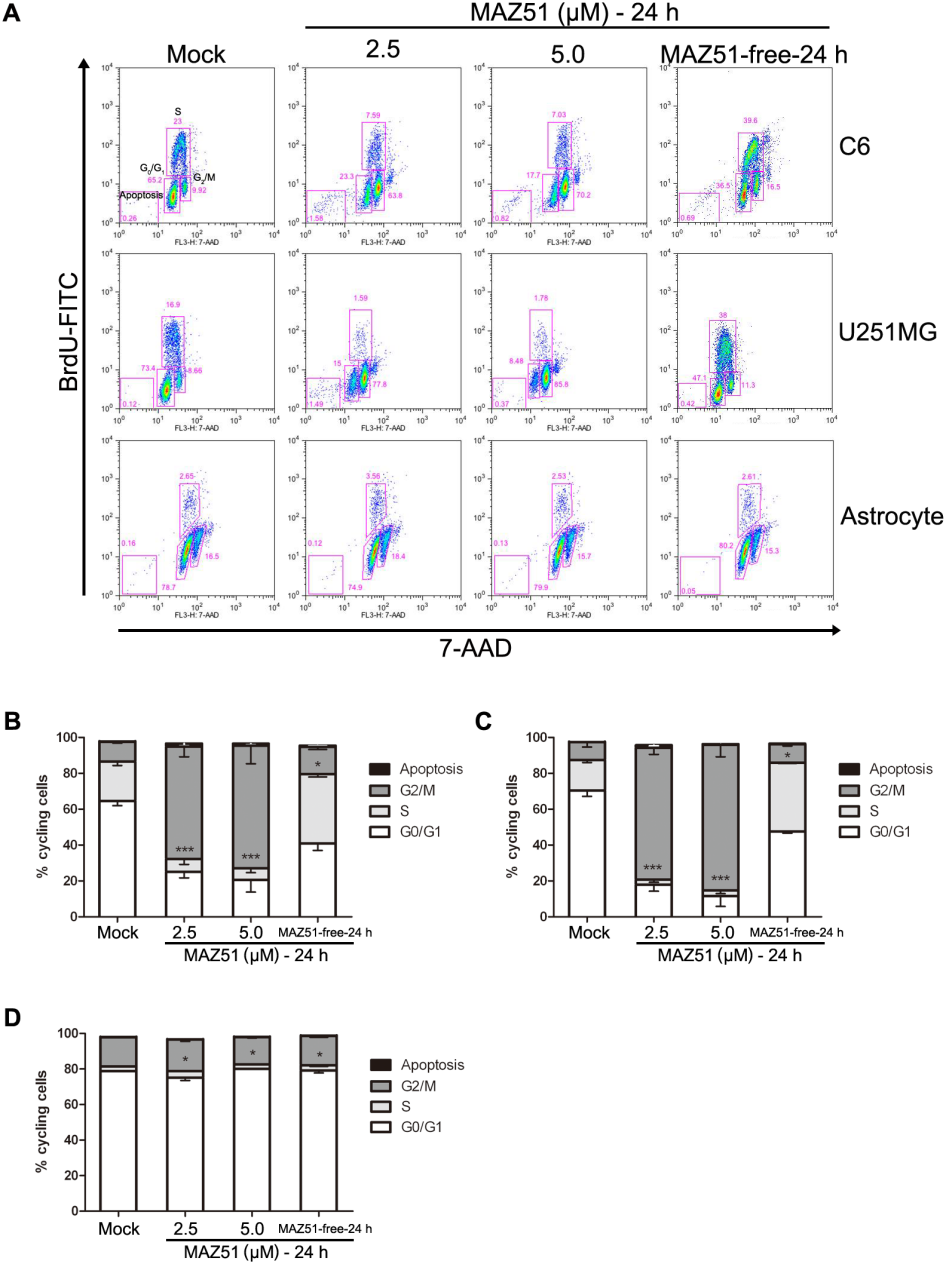
MAZ51 reversibly causes G2/M phase arrest in glioma cells but not in primary astrocytes. (A) Cells were treated for 24 h either in the absence (control) or presence of MAZ51 (2.5 µM or 5.0 µM). Cells were also treated with 5.0 µM MAZ51 for 24 h and then incubated for an additional 24 h in fresh medium without MAZ51 (MAZ51-free-24 h). Cell cycle distribution was analyzed by flow cytometry after coupled staining with a fluorescein isothiocyanate (FITC)-conjugated anti-BrdU antibody and 7-amino-actinomycin D (7-AAD). (B–D) Cell cycle distribution is expressed as the percentage of cells in each cell cycle phase for C6 cells (B), U251MG cells (C), and rat primary astrocytes (D). Data are representative of three independent experiments. Statistical significance was determined by two-way ANOVA followed by the Bonferroni multiple comparison test using GraphPad Prism. ***P<0.001; **P<0.01; *P<0.05.

To investigate whether the morphological alteration caused by MAZ51 correlated with its cytotoxicity and effects on cell cycle progression, a cell cycle assay using flow cytometry was performed in C6 and U251MG glioma cells, and primary astrocytes. The flow cytometry analysis revealed a dose-dependent increase of the G2/M cell population in both C6 and U251MG cells treated with MAZ51 (Fig. 2). Culturing of C6 cells for 24 h in the presence of MAZ51 greatly increased the proportion of these cells in the G2/M phase of the cell cycle (9.92%±0.16% in control cells; 63.8%±1.73% in cells treated with 2.5 µM MAZ51; 70.2%±1.78% in cells treated with 5.0 µM MAZ51). There was a concomitant decrease in the proportion of these cells in the G0/G1 phase (65.2%±0.38% in control cells; 23.3%±0.73% in cells treated with 2.5 µM MAZ51; 17.7%±0.61% in cells treated with 5.0 µM MAZ51) (Figs. 2A, B). Results obtained with U251MG cells were similar, although MAZ51 treatment was more effective with control cells in this line: 8.66% of control cells were in the G2/M phase, but 77.8% of cells treated with 2.5 µM MAZ51 and 85.8% of cells treated with 5.0 µM MAZ51 were in the G2/M phase, 24 h after the treatment (Figs. 2A, C). In spite of the effects of MAZ51 on the cell cycle, this compound did not induce a significant decrease in cell viability in glioma cell lines by 24 h of treatment. In addition, the G2/M arrest of glioma cells by MAZ51 was reversed when MAZ51-containing medium was replaced with fresh medium without MAZ51 (Fig. 2A). In agreement with the morphological data, no significant alteration in the cell cycle pattern was observed in primary astrocytes treated with up to 5.0 µM MAZ51. This suggests that MAZ51 preferentially targets cancer cells (Figs. 2A, D).

### The effects of MAZ51 in C6 glioma cells are related to the effects on the Akt/GSK3β and Rho signaling pathways

**Figure 3:**
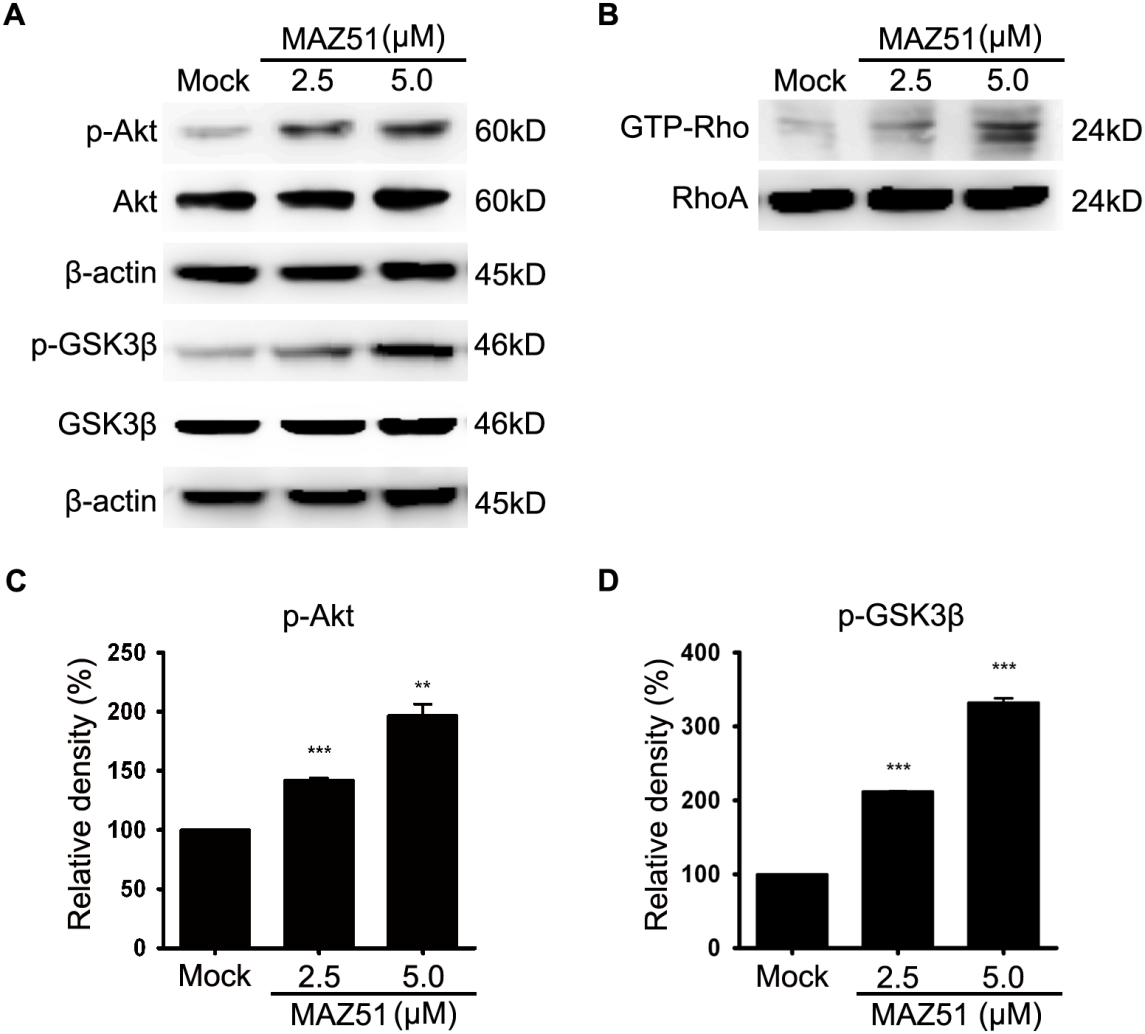
The effects of MAZ51 in C6 glioma cells are related to changes in the Akt/GSK3β and RhoA pathways. (A) Western blot analysis of GSK3β, p-GSK3β (Ser9), Akt, p-Akt (Ser473), and β-actin in C6 cells treated with 2.5 µM or 5.0 µM MAZ51 for 24 h. Controls were treated with 0.1% DMSO. (B) Expressions of active GTP-bound RhoA and total RhoA in C6 cells treated with 2.5 µM or 5.0 µM MAZ51 for 24 h. GTP-bound RhoA was extracted with the Rhotekin pull-down assay. (C, D) Densitometric analyses of western blots. Statistical significance was determined by one-way ANOVA followed by the Bonferroni multiple comparison tests using GraphPad Prism. Values are expressed as percentage of control and were obtained from three independent experiments; each independent experiment was performed in triplicate. ***P<0.001; **P<0.01.

PI3K/Akt, and the Akt downstream target GSK3β, are important in regulating cell morphology and cell migration in several cell types, including glioma cells [19]–[21]. Thus, we examined whether MAZ51 could influence Akt phosphorylation status and the activation or inactivation profiles of GSK3β in C6 glioma cells. Treatment of C6 cells with MAZ51 resulted in a dose-dependent increase in Akt phosphorylation without affecting total Akt levels (Figs. 3A, C). We then examined whether GSK3β was also affected by MAZ51. MAZ51 treatment increased the phosphorylation of GSK3β at the Ser9 residue, which represents the inactive form of GSK3β, without changing the total levels of GSK3β (Figs. 3A, D). These data indicate that treatment of C6 glioma cells with MAZ51 increased Akt activity with resultant inactivation of GSK3β.

RhoA, one of best-characterized Rho-GTPases, has a key role in regulating the dynamics of actin filaments [22]. Given the morphological and cytoskeletal alterations that are characteristic of RhoA activation (Fig. 1), we investigated whether MAZ51 affected the activity of RhoA in C6 cells. Treatment of C6 cells with MAZ51 induced dose-dependent increases in the amount of active, GTP-bound RhoA, but did not alter the total RhoA amount (Fig. 3B). This indicated that RhoA activation occurs during the observed morphological and cytoskeletal alterations in C6 cells treated with MAZ51.

### The effects of MAZ51 in C6 glioma cells are independent of the inhibition of VEGFR-3 phosphorylation

**Figure 4:**
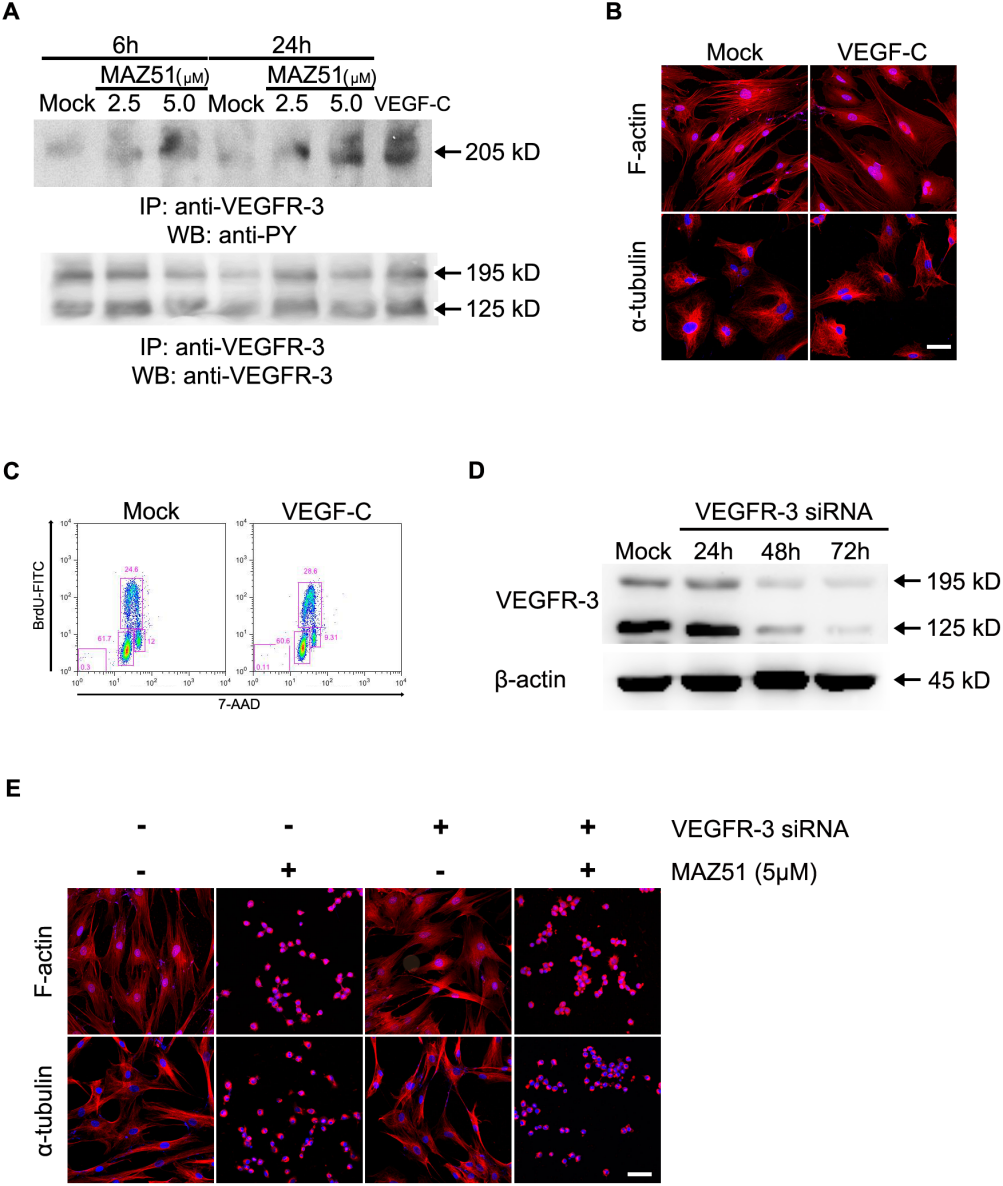
The effects of MAZ51 in C6 cells are independent of the inhibition of VEGFR-3 phosphorylation. (A) C6 cells were treated with 2.5 µM or 5.0 µM MAZ51 for 6 h or 24 h. In parallel, cells were treated with recombinant rat VEGF-C protein (150 ng/mL) for 20 min. Cell lysates were immunoprecipitated with anti-VEGFR-3 antibody and subsequently assayed by western blot analysis using an anti-phosphotyrosine antibody. The loading control is represented by the western blot of VEGFR-3 with the double bands indicating the larger glycosylated (195 kD) and smaller cleaved (125 kD) forms. Effects of treatment with VEGF-C on cytoskeletal rearrangements (B) and cell cycle distribution (C) of C6 cells. C6 cells were treated with VEGF-C (150 ng/mL) for 20 min, while control cells were treated with 0.1% DMSO. (D) Transient transfection using VEGFR-3 siRNA or scrambled RNA (mock) for the indicated time periods. C6 cells transfected with VEGFR-3 siRNA exhibited the most extensive knockdown of the protein level at 72 h after transfection. (E) Effects of 5 µM MAZ51 on the morphological and cytoskeletal rearrangements of C6 cells after transfection with VEGFR-3 siRNA or scrambled siRNA. Data are representative of three independent experiments. Scale bar = 50 µm.

Because MAZ51 inhibits tyrosine phosphorylation of VEGFR-3 in endothelial cell lines [2], [8], [9], we examined whether the effects of MAZ51 observed in C6 cells depend on the inhibition of VEGFR-3 phosphorylation. Immunoprecipitation-western blot analysis using C6 glioma cells revealed that MAZ51 appeared to increase, rather than decrease, tyrosine phosphorylation of VEGFR-3 (Fig. 4A), while VEGF-C induced the tyrosine phosphorylation of VEGFR-3. Although these results were confirmed through repetition, confidence in this finding was increased through several additional experiments using C6 cells. First, enhancing VEGFR-3 phosphorylation by the addition of VEGF-C had no effect on cell shape or cytoskeleton arrangements, such as rearrangements involving F-actin and microtubules (Fig. 4B). In addition, no significant alterations in the cell cycle pattern were observed in C6 cells treated with VEGF-C (Fig. 4C). These results suggest that MAZ51 causes alterations in the cytoskeletal arrangement and the cell cycle pattern of glioma cells independent of the tyrosine phosphorylation of VEGFR-3.

To provide additional evidence supporting this conclusion, we tested whether decreasing the expression of VEGFR-3 in C6 cells causes morphological and cytoskeletal alterations, and whether the observed effects of MAZ51 also occur in VEGFR-3-silenced C6 cells. At 48 h and 72 h after transfection of C6 cells with VEGFR-3 siRNA, there was a significant decrease in VEGFR-3 mRNA and protein levels; transfection of C6 cells with control siRNA had no effect on VEGFR-3 expression (Fig. 4D and Figure S1). Silencing of VEGFR-3 did not cause any changes in cell shape or cytoskeletal arrangements (Fig. 4E), and did not induce any alterations in cell cycle patterns (data not shown). In addition, when treated with MAZ51, C6 cells with decreased expression of VEGFR-3 showed the same effects as control cells, including a significant loss of both actin stress fibers and microtubule networks (Fig. 4E). Considered together, these data suggest that the inhibition of VEGFR-3 phosphorylation is not required for the effects of MAZ51 in C6 glioma cells.

## Discussion

Indolinones, such as MAZ51, inhibit the activity of receptor tyrosine kinases, and thus have potent anti-angiogenic effects and direct antitumor activities [1]. The key finding of the current study is that MAZ51 disrupts the cytoskeletal architecture, including actin filaments and microtubules, in C6 and U251MG glioma cells, causing rapid changes in cell morphology, with retracting processes, and eventually cell rounding. MAZ51-induced morphological changes, such as cell shrinkage, loss of adherence, and cell rounding, are common features of cells undergoing apoptosis. However, MAZ51-induced morphological and cytoskeletal alterations occurred at concentrations that did not cause significant cell death. In addition, when glioma cells treated with MAZ51 were placed in fresh medium without MAZ51, the MAZ51-induced morphological changes were reversed. Thus, the alterations in cell shape induced by MAZ51 were not due to apoptosis or necrosis. Given that actin filaments and the microtubule cytoskeleton are key components involved in cell migration, MAZ51-induced disruption of the F-actin and microtubule cytoskeleton in glioma cells presumably affects their adhesive properties and migratory ability.

Interestingly, flow cytometric analysis revealed that MAZ51 caused a significant dose- and time-dependent accumulation of C6 and U251MG cells in the G2/M phase. Removal of MAZ51 led to nearly normal cell cycle progression in cells arrested in G2/M phase, indicating that the MAZ51-induced G2/M phase arrest is reversible. The dynamics of the actin cytoskeleton are involved in controlling cell shape and cell migration and are critical during cell cycle progression and mitosis [22]. Thus, our data suggest a possible link between cell cycle arrest and cytoskeletal alterations, both of which occurred in glioma cells in similar dose- and time-dependent patterns in response to treatment with MAZ51.

Mitotic cell rounding is the process in which flat interphase cells retract their margins to reduce their spread area, taking on a near-spherical shape; this process is accompanied by dramatic remodeling of the actin cytoskeleton, generating a rigid and rounded actomyosin cortex [23]–[27]. In this regard, the morphological and cytoskeletal alterations caused by MAZ51 could be attributed to G2/M phase arrest. In support of this, our data show a MAZ51-induced, dose-dependent increase of GTP-bound RhoA in C6 cells. This finding is consistent with those of previous studies, which showed that increases in the activity of RhoA and the resulting activation of its effector, the Rho associated kinase ROCK, induce remodeling of actomyosin; this drives both mitotic rounding and cortical stiffening [25], [28], [29]. However, considering that RhoA is the key regulator of actin cytoskeleton dynamics involved in stress fiber formation, cell rounding, and retraction of cell processes [22], [30]–[36], it remains to be determined whether the cell rounding induced by MAZ51 is caused by the persistent mitotic cell rounding due to G2/M phase arrest.

PI3K/Akt, and the Akt downstream target GSK3β, have been implicated in actin filament remodeling and cell migration in several cell types, including glioma cells [19]–[21], [37], [38]. Activation of Akt results in increased phosphorylation of GSK3β at Ser9, which prevents Rac1-mediated migration by stabilizing the actin cortex [37]. In addition, Rac1 activation is essential for astrocyte stellation and migration, which results from the restructuring of the cytoskeleton; this process involves the rearrangement of both microtubules and microfilaments, and can lead to the outgrowth of processes with lamellipodia [39], [40]. Interestingly, we found that MAZ51 activated Akt and increased the phosphorylation of GSK3β at position Ser9, which represents the inactive form of GSK3β; this suggests that Akt-mediated inhibition of GSK3β may contribute to the cytoskeletal alterations in glioma cells induced by MAZ51. Considered together, our data suggest that the effects of MAZ51 in glioma cells could be mediated through phosphorylation of Akt/GSK3β and activation of RhoA. However, further studies are required to determine whether other signaling pathways are involved in the effects of MAZ51.

It is noteworthy that MAZ51 has tumor-selectivity in its ability to cause cytoskeletal and cell cycle alterations. We found that the effects of MAZ51 seen in C6 and U251MG cells also occur in human glioma cell lines, U87MG and T98G, both of which became round and retractile after MAZ51 treatment (unpublished data). However, in rat primary cortical astrocytes, MAZ51 had no effect on the cell-cycle pattern or the morphology and cytoskeleton arrangements, at the same concentrations that affected transformed cells. Several indolinone derivatives, including sunitinib and toceranib, showed potential anti-proliferative activity in cancer cell lines, suggesting the potential therapeutic application of indolinones as antitumor agents [2]–[7]. Overall, our data suggest that MAZ51 exhibits anti-proliferative and possibly anti-migratory effects selectively against glioma cell lines, similar to other indolinone derivatives.

In the present study, MAZ51 did not inhibit tyrosine phosphorylation of VEGFR-3, but rather increased its phosphorylation. This was in contrast to the inhibition of VEGFR-3 phosphorylation seen with MAZ51 in several other cell lines, including endothelial cells [2], [3], [8], [9]. Thus, several additional experiments were conducted to enhance confidence in these unanticipated results. First, as a positive control for immunoprecipitation-western blot analyses, VEGF-C was shown to enhance the tyrosine phosphorylation of VEGFR-3 in glioma cells. This is consistent with previous reports showing that VEGF-C induces the tyrosine phosphorylation of VEGFR-3. Second, treatment of glioma cells with VEGF-C affected neither cytoskeleton arrangements nor cell cycle patterns, despite its ability to increase tyrosine phosphorylation of VEGFR-3. Third, decreasing the expression of VEGFR-3 in glioma cells did not cause any morphological or cytoskeletal alterations and did not induce any alterations in the cell cycle patterns. Finally, MAZ51 treatment of C6 cells with decreased VEGFR-3 expression caused the same alterations of morphology and cytoskeletal arrangements as in control cells. Thus, although the reason for this discrepancy is unclear, it is reasonable to conclude that the effect of MAZ51 in C6 glioma cells does not require the inhibition of VEGFR-3 phosphorylation.

Recently, it was shown that VEGF-C provokes the rearrangement of actin filaments in cervical cancer cells and drives cell migration and invasion via upregulation of galectin-3 protein and subsequent VEGFR-3 activation [41], [42]. Because previous reports used endothelial cells [2], [8], [9] or oral squamoid cancer cells [3], the discrepancy in the effect of MAZ51 on VEGFR-3 phosphorylation in glioma cells could be due to differences in cell lines. However, further studies are needed to determine the precise mechanism(s) for MAZ51-induced alterations in glioma cell lines.

In conclusion, using two glioma cell lines, our data have shown the following: 1) MAZ51 causes dramatic shape changes due to the clustering and aggregation of F-actin and microtubules, 2) MAZ51 induces cell cycle arrest at the G2/M phase in rat glioma C6 and human glioma U251MG cell lines, 3) MAZ51 selectively targets transformed cells but not primary astrocytes, 4) Akt-mediated inhibition of GSK3β and activation of Rho are involved in the effects of MAZ51, and 5) the effects of MAZ51 are independent of the inhibition of VEGFR-3 phosphorylation in rat glioma C6 cells. Although the precise mechanism(s) for MAZ51-induced cell rounding and cell cycle G2/M arrest are not clear at the present time, our data indicate that MAZ51 treatment inhibits proliferation, and presumably migration, of glioma cells, suggesting that MAZ51 is a candidate for further clinical investigation for treating gliomas.

## Supporting Information

Figure S1 **PCR amplification and quantitative real-time reverse transcriptase-polymerase chain reaction (qRT-PCR) for VEGFR-3 mRNA in C6 cells transiently transfected with VEGFR-3 siRNA or scrambled RNA for the indicated time periods.** (A) Oligonucleotide primers used in PCR amplification and qRT-PCR; primer pairs originated from rat VEGFR-3 mRNA. (B) VEGFR-3 mRNA expression was determined after transfection using VEGFR-3 siRNA or scrambled RNA (mock); the mock control was collected at 72 h post-transfection. Data are representative of three independent experiments. (C) The expression level of VEGFR-3 was calculated using the comparative threshold cycle method (2^−ΔΔCt^) with *GAPDH* as the control gene. Statistical significance was determined by one-way ANOVA followed by the Bonferroni multiple comparison test using GraphPad Prism. ***P<0.001; **P<0.01.
(TIF)

## References

[1] Prakash CR, Theivendren P, Raja S (2012) Indolin-2-Ones in Clinical Trials as Potential Kinase Inhibitors: A Review. Pharmacology & Pharmacy 3.

[2] Kirkin V, Mazitschek R, Krishnan J, Steffen A, Waltenberger J, et al (2001) Characterization of indolinones which preferentially inhibit VEGF-C- and VEGF-D-induced activation of VEGFR-3 rather than VEGFR-2. Eur J Biochem 268: 5530–5540. 11683876 10.1046/j.1432-1033.2001.02476.x

[3] Matsuura M, Onimaru M, Yonemitsu Y, Suzuki H, Nakano T, et al (2009) Autocrine Loop between Vascular Endothelial Growth Factor (VEGF)-C and VEGF Receptor-3 Positively Regulates Tumor-Associated Lymphangiogenesis in Oral Squamoid Cancer Cells. The American Journal of Pathology 175: 1709–1721. 19779139 10.2353/ajpath.2009.081139 PMC2751566

[4] Cho TP, Dong SY, Jun F, Hong FJ, Liang YJ, et al (2010) Novel potent orally active multitargeted receptor tyrosine kinase inhibitors: synthesis, structure-activity relationships, and antitumor activities of 2-indolinone derivatives. J Med Chem 53: 8140–8149. 21028894 10.1021/jm101036c

[5] Xiong X, Zhang Y, Gao X, Dong Z, Li L, et al (2010) B5, a novel pyrrole-substituted indolinone, exerts potent antitumor efficacy through G2/M cell cycle arrest. Invest New Drugs 28: 26–34. 19139818 10.1007/s10637-008-9211-7

[6] Zou H, Zhang L, Ouyang J, Giulianotti MA, Yu Y (2011) Synthesis and biological evaluation of 2-indolinone derivatives as potential antitumor agents. Eur J Med Chem 46: 5970–5977. 22019188 10.1016/j.ejmech.2011.10.009

[7] Bisping G, Kropff M, Wenning D, Dreyer B, Bessonov S, et al (2006) Targeting receptor kinases by a novel indolinone derivative in multiple myeloma: abrogation of stroma-derived interleukin-6 secretion and induction of apoptosis in cytogenetically defined subgroups. Blood 107: 2079–2089. 16278310 10.1182/blood-2004-11-4250

[8] Kirkin V, Thiele W, Baumann P, Mazitschek R, Rohde K, et al (2004) MAZ51, an indolinone that inhibits endothelial cell and tumor cell growth in vitro, suppresses tumor growth in vivo. Int J Cancer 112: 986–993. 15386354 10.1002/ijc.20509

[9] Benedito R, Rocha SF, Woeste M, Zamykal M, Radtke F, et al (2012) Notch-dependent VEGFR3 upregulation allows angiogenesis without VEGF-VEGFR2 signalling. Nature 484: 110–114. 22426001 10.1038/nature10908

[10] Lee JY, Park S, Kim DC, Yoon JH, Shin SH, et al (2013) A VEGFR-3 antagonist increases IFN-gamma expression on low functioning NK cells in acute myeloid leukemia. J Clin Immunol 33: 826–837. 23404187 10.1007/s10875-013-9877-2

[11] DeAngelis LM (2001) Brain tumors. N Engl J Med 344: 114–123. 11150363 10.1056/NEJM200101113440207

[12] Wen PY, Kesari S (2008) Malignant gliomas in adults. N Engl J Med 359: 492–507. 18669428 10.1056/NEJMra0708126

[13] Veikkola T, Jussila L, Makinen T, Karpanen T, Jeltsch M, et al (2001) Signalling via vascular endothelial growth factor receptor-3 is sufficient for lymphangiogenesis in transgenic mice. Embo j 20: 1223–1231. 11250889 10.1093/emboj/20.6.1223 PMC145532

[14] Karkkainen MJ, Haiko P, Sainio K, Partanen J, Taipale J, et al (2004) Vascular endothelial growth factor C is required for sprouting of the first lymphatic vessels from embryonic veins. Nat Immunol 5: 74–80. 14634646 10.1038/ni1013

[15] Jenny B, Harrison JA, Baetens D, Tille JC, Burkhardt K, et al (2006) Expression and localization of VEGF-C and VEGFR-3 in glioblastomas and haemangioblastomas. J Pathol 209: 34–43. 16523449 10.1002/path.1943

[16] Grau SJ, Trillsch F, Herms J, Thon N, Nelson PJ, et al (2007) Expression of VEGFR3 in glioma endothelium correlates with tumor grade. J Neurooncol 82: 141–150. 17115285 10.1007/s11060-006-9272-4

[17] Auer RN, Del Maestro RF, Anderson R (1981) A simple and reproducible experimental in vivo glioma model. Can J Neurol Sci 8: 325–331. 7326613 10.1017/s0317167100043468

[18] Kazenwadel J, Secker GA, Betterman KL, Harvey NL (2012) In vitro assays using primary embryonic mouse lymphatic endothelial cells uncover key roles for FGFR1 signalling in lymphangiogenesis. PLoS One 7: e40497. 22792354 10.1371/journal.pone.0040497 PMC3391274

[19] Farooqui R, Zhu S, Fenteany G (2006) Glycogen synthase kinase-3 acts upstream of ADP-ribosylation factor 6 and Rac1 to regulate epithelial cell migration. Exp Cell Res 312: 1514–1525. 16529739 10.1016/j.yexcr.2006.01.018

[20] Brozzi F, Arcuri C, Giambanco I, Donato R (2009) S100B Protein Regulates Astrocyte Shape and Migration via Interaction with Src Kinase: Implications for Astrocyte Development, Activation, and Tumor Growth. J Biol Chem 284: 8797–8811. 19147496 10.1074/jbc.M805897200 PMC2659238

[21] Atkins RJ, Dimou J, Paradiso L, Morokoff AP, Kaye AH, et al (2012) Regulation of glycogen synthase kinase-3 beta (GSK-3beta) by the Akt pathway in gliomas. J Clin Neurosci 19: 1558–1563. 22999562 10.1016/j.jocn.2012.07.002

[22] Klopocka W, Korczynski J, Pomorski P (2013) Cytoskeleton and nucleotide signaling in glioma C6 cells. Adv Exp Med Biol 986: 103–119. 22879066 10.1007/978-94-007-4719-7_6

[23] Cramer LP, Mitchison TJ (1997) Investigation of the mechanism of retraction of the cell margin and rearward flow of nodules during mitotic cell rounding. Mol Biol Cell 8: 109–119. 9017599 10.1091/mbc.8.1.109 PMC276063

[24] Kunda P, Pelling AE, Liu T, Baum B (2008) Moesin controls cortical rigidity, cell rounding, and spindle morphogenesis during mitosis. Curr Biol 18: 91–101. 18207738 10.1016/j.cub.2007.12.051

[25] Matthews HK, Delabre U, Rohn JL, Guck J, Kunda P, et al (2012) Changes in Ect2 localization couple actomyosin-dependent cell shape changes to mitotic progression. Dev Cell 23: 371–383. 22898780 10.1016/j.devcel.2012.06.003 PMC3763371

[26] Matthews HK, Baum B (2012) The metastatic cancer cell cortex: an adaptation to enhance robust cell division in novel environments? Bioessays 34: 1017–1020. 22996605 10.1002/bies.201200109

[27] Lancaster OM, Baum B (2014) Shaping up to divide: Coordinating actin and microtubule cytoskeletal remodelling during mitosis. Semin Cell Dev Biol. 10.1016/j.semcdb.2014.02.015 24607328

[28] Maddox AS, Burridge K (2003) RhoA is required for cortical retraction and rigidity during mitotic cell rounding. J Cell Biol 160: 255–265. 12538643 10.1083/jcb.200207130 PMC2172639

[29] David B (2012) Mechanics of the Cell.

[30] Hall A (1998) Rho GTPases and the actin cytoskeleton. Science 279: 509–514. 9438836 10.1126/science.279.5350.509

[31] Kalman D, Gomperts SN, Hardy S, Kitamura M, Bishop JM (1999) Ras family GTPases control growth of astrocyte processes. Mol Biol Cell 10: 1665–1683. 10233170 10.1091/mbc.10.5.1665 PMC30489

[32] Majumdar M, Seasholtz TM, Goldstein D, de Lanerolle P, Brown JH (1998) Requirement for Rho-mediated myosin light chain phosphorylation in thrombin-stimulated cell rounding and its dissociation from mitogenesis. J Biol Chem 273: 10099–10106. 9553056 10.1074/jbc.273.17.10099

[33] Zhou YT, Guy GR, Low BC (2006) BNIP-Salpha induces cell rounding and apoptosis by displacing p50RhoGAP and facilitating RhoA activation via its unique motifs in the BNIP-2 and Cdc42GAP homology domain. Oncogene 25: 2393–2408. 16331259 10.1038/sj.onc.1209274

[34] Tas PW, Gambaryan S, Roewer N (2007) Volatile anesthetics affect the morphology of rat glioma C6 cells via RhoA, ERK, and Akt activation. J Cell Biochem 102: 368–376. 17492663 10.1002/jcb.21294

[35] Maeda M, Hasegawa H, Hyodo T, Ito S, Asano E, et al (2011) ARHGAP18, a GTPase-activating protein for RhoA, controls cell shape, spreading, and motility. Mol Biol Cell 22: 3840–3852. 21865595 10.1091/mbc.E11-04-0364 PMC3192863

[36] Rodnight RB, Gottfried C (2013) Morphological plasticity of rodent astroglia. J Neurochem 124: 263–275. 23278277 10.1111/jnc.12087

[37] Vaidya RJ, Ray RM, Johnson LR (2006) Akt-mediated GSK-3beta inhibition prevents migration of polyamine-depleted intestinal epithelial cells via Rac1. Cell Mol Life Sci 63: 2871–2879. 17109063 10.1007/s00018-006-6379-x PMC11136338

[38] Karrasch T, Spaeth T, Allard B, Jobin C (2011) PI3K-dependent GSK3ss(Ser9)-phosphorylation is implicated in the intestinal epithelial cell wound-healing response. PLoS One 6: e26340. 22039465 10.1371/journal.pone.0026340 PMC3198390

[39] Seasholtz TM, Radeff-Huang J, Sagi SA, Matteo R, Weems JM, et al (2004) Rho-mediated cytoskeletal rearrangement in response to LPA is functionally antagonized by Rac1 and PIP2. Journal of Neurochemistry 91: 501–512. 15447683 10.1111/j.1471-4159.2004.02749.x

[40] Racchetti G, D’Alessandro R, Meldolesi J (2012) Astrocyte stellation, a process dependent on Rac1 is sustained by the regulated exocytosis of enlargeosomes. Glia 60: 465–475. 22144092 10.1002/glia.22280 PMC3306795

[41] He M, Cheng Y, Li W, Liu Q, Liu J, et al (2010) Vascular endothelial growth factor C promotes cervical cancer metastasis via up-regulation and activation of RhoA/ROCK-2/moesin cascade. BMC Cancer 10: 170. 20429915 10.1186/1471-2407-10-170 PMC2873393

[42] Liu J, Cheng Y, He M, Yao S (2014) Vascular endothelial growth factor C enhances cervical cancer cell invasiveness via upregulation of galectin-3 protein. Gynecol Endocrinol. 10.3109/09513590.2014.898054 24650367

