# Supplementary figures and images for: The Indolinone MAZ51 Induces Cell Rounding and G2/M Cell Cycle Arrest in Glioma Cells without the Inhibition of VEGFR-3 Phosphorylation: Involvement of the RhoA and Akt/GSK3β Signaling Pathways
Source: PLoS One. 2014 Sep 30;9(9):e109055. doi: 10.1371/journal.pone.0109055 (PMC4182637; doi:10.1371/journal.pone.0109055)

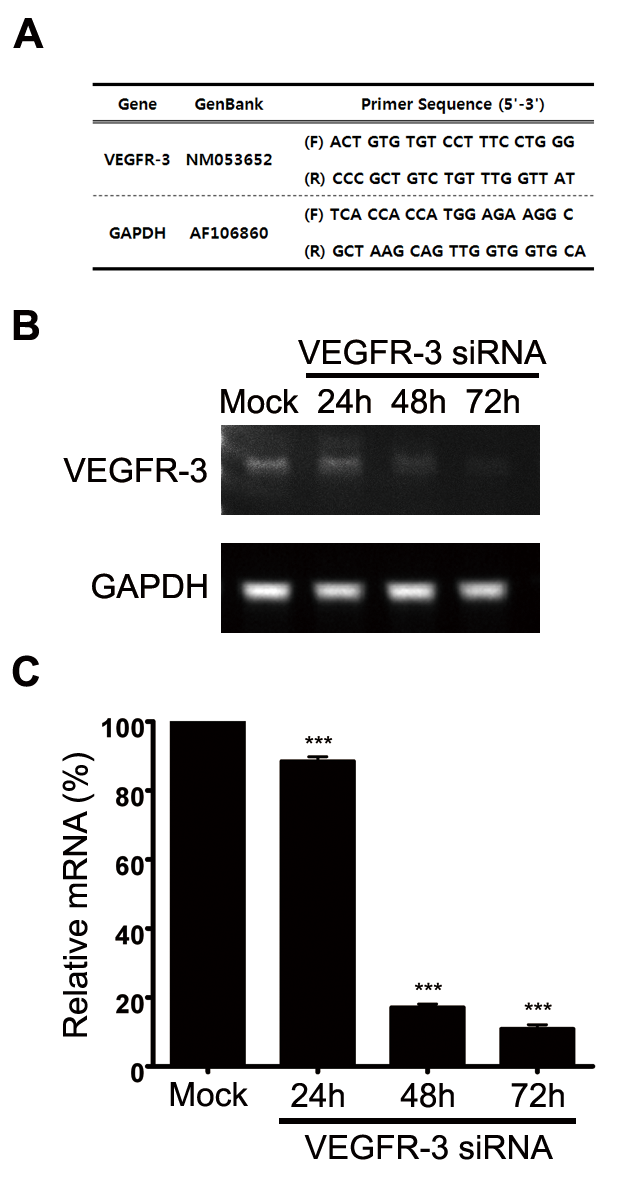

Supplement: Figure S1 — PCR amplification and quantitative real-time reverse transcriptase-polymerase chain reaction (qRT-PCR) for VEGFR-3 mRNA in C6 cells transiently transfected with VEGFR-3 siRNA or scrambled RNA for the indicated time periods. (A) Oligonucleotide primers used in PCR amplification and qRT-PCR; primer pairs originated from rat VEGFR-3 mRNA. (B) VEGFR-3 mRNA expression was determined after transfection using VEGFR-3 siRNA or scrambled RNA (mock); the mock control was collected at 72 h post-transfection. Data are representative of three independent experiments. (C) The expression level of VEGFR-3 was calculated using the comparative threshold cycle method (2−ΔΔCt) with GAPDH as the control gene. Statistical significance was determined by one-way ANOVA followed by the Bonferroni multiple comparison test using GraphPad Prism. ***P<0.001; **P<0.01. (TIF) [file pone.0109055.s001.tif]
